# Initial management of newly diagnosed WHO grade 2–3 adult meningioma following surgery: results from the Dutch Brain Tumour Registry (2016–2021)

**DOI:** 10.1007/s11060-024-04730-2

**Published:** 2024-08-29

**Authors:** Vincent K.Y. Ho, Monique M. Anten, Anniek Garst, Eelke M. Bos, Tom J. Snijders, Daniëlle B.P. Eekers, Tatjana Seute, the Dutch Brain Tumour Registry (DBTR) and the Dutch Society for Neuro-Oncology (LWNO)

**Affiliations:** 1 Department of Research and Development, Netherlands Comprehensive Cancer Organisation (IKNL), P.O. Box 19079, 3501 DB Utrecht, The Netherlands; 2 Department of Neurology and Neurosurgery, University Medical Centre Utrecht, Utrecht, The Netherlands; 3 Department of Neurology, Maastricht University Medical Centre+, Maastricht, The Netherlands; 4 Department of Neurosurgery, Erasmus MC Cancer Institute, University Medical Centre Rotterdam, Maastricht, The Netherlands; 5 Department of Radiation Oncology (Maastro), GROW School for Oncology and Reproduction, Maastricht University Medical Centre+, Maastricht, The Netherlands

**Keywords:** Meningioma, Surgery, Radiotherapy, Population-based registry

## Abstract

**Purpose:**

Meningiomas classified as grade 2–3 according to the World Health Organisation (WHO) require combined surgery and in most cases radiotherapy (RT). Their initial management was evaluated using the Dutch Brain Tumour Registry.

**Methods:**

The study included 393 patients aged ≥ 18 years with newly diagnosed meningioma WHO grade 2–3 between 2016 and 2021. Factors associated with adjuvant RT < 6 months following surgery were identified using logistic regression analyses, thereby accounting for variation between CNS regional tumour boards through mixed-effect modelling. This variation was further assessed by funnel plots for case-mix adjusted ratios of RT across tumour boards. The association with patients’ survival at 5 years was evaluated with inverse probability-weighted accelerated failure (Weibull) models. Analyses were performed on multiple imputed datasets (m = 10) to account for missing data.

**Results:**

Adjuvant RT was administered to 22.2% (59/266) of patients with WHO grade 2 meningioma following a total resection, to 61.1% (58/95) following a partial resection, and to 68.8% (22/32) of patients with WHO grade 3 meningioma (61.5% after partial and 73.7% after total resection). RT was associated with grade 3, partial resection, bone invasion, and absence of multiple lesions. Management varied across tumour boards for grade 2 meningioma following total resection. Adjuvant RT was associated with survival benefit in case of grade 3 disease (hazard ratio: 0.40, 95%-confidence interval: 0.16–0.95, *p* = 0.04).

**Conclusion:**

This national review revealed variation across CNS regional tumour boards in the management of grade 2 meningioma following total resection, and demonstrated survival benefit of adjuvant RT in grade 3 meningioma.

**Supplementary Information:**

The online version contains supplementary material available at 10.1007/s11060-024-04730-2.

## Introduction

Although the majority of meningiomas are grade 1 and considered benign, those classified as grade 2 (atypical) and grade 3 (malignant) according to the World Health Organisation (WHO) are associated with an increased risk of recurrence, invasion into the brain parenchyma, and inferior prognosis [1]. Together, these tumours comprise around 10% of all newly histologically confirmed meningiomas. Relative survival after 10 years was recently estimated at 71.3% for patients with WHO grade 2, and 36.4% for those with WHO grade 3 meningioma [2].

Fractionated external beam radiotherapy (RT) following surgery forms a mainstay in the management of WHO grade 2–3 meningiomas, depending on their risk profile. According to the guidelines issued by the European Association of Neuro-Oncology (EANO), adjuvant RT is recommended for high-risk tumours, which include WHO grade 3 meningiomas, recurrent WHO grade 2 tumours, and WHO grade 2 tumours that are not totally removed by surgery [3]. WHO grade 2 tumours removed by gross total resection are considered intermediate-risk and may be managed by either RT or watchful waiting, depending on their resectability in case of a recurrence.

Aside from several large retrospective studies [4, 5], the benefits of adjuvant RT have been substantiated by prospective phase II trials by the Radiation Therapy Oncology Group (RTOG) and the European Organisation for Research and Treatment of Cancer (EORTC). In RTOG 0539, patients with intermediate-risk meningiomas (which also included recurrent WHO grade I tumours) achieved a 3-year progression-free survival (PFS) and overall survival (OS) of 94% and 96%, respectively [6]. In those with high-risk disease, adjuvant RT resulted in a 3-year PFS of 59%, and OS of 79% [7]. In EORTC 22,042, corresponding rates were 89% and 98% among patients with a WHO grade 2 tumour following total resection [8].

Whether WHO grade 2 meningiomas that are removed in toto should subsequently be managed by (early) RT or observation remains subject to debate. Although most studies report advantage of RT regarding PFS [9, 10], superiority in terms of OS remains unclear [11, 12]. By postponing treatment, patients may initially be spared some of the potential adverse effects associated with RT, including neurocognitive decline, hypopituitarism, and an increased risk of developing secondary tumours. More clarity on this matter is anticipated with the completion of two prospective controlled phase III trials, the recently closed ROAM/EORTC 1308 trial [13]—with results being expected for 2025—and the ongoing NRG-BN003 trial (ClinicalTrials.gov Identifier: NCT03180268).

Until the role of early adjuvant RT may be better defined, consensus on the standard of care is difficult to establish. Therefore, the options of treatment or active monitoring are largely left to be discussed on an individual basis and in multidisciplinary tumour boards. As a result of this, administration of adjuvant RT shows considerable variation across patients and healthcare providers [14, 15].

This study reports on the initial management of new WHO grade 2–3 meningioma in the Netherlands, contributing to the evidence base beyond clinical trials. Using the population-based Dutch Brain Tumour Registry (DBTR), we specifically assessed the use of RT < 6 months after surgery in adult patients diagnosed between 2016 and 2021, also aiming to discern factors associated with early adjuvant RT. We thereby accounted for variation across regional tumour boards, which represent networks of hospitals cooperating in the diagnosis, treatment, and care for patients with central nervous system (CNS) tumours.

## Methods

### Data source

The Dutch Brain Tumour Registry (DBTR) is an expansion of the Netherlands Cancer Registry (NCR) for tumours of the CNS. This nationwide population-based registry is hosted by the Netherlands Comprehensive Cancer Organisation (Integraal Kankercentrum Nederland [IKNL]), and includes 90–95% of malignant diseases [16]. For meningiomas, completeness is estimated at 98% for histologically confirmed tumours [2].

Information on patient, tumour and treatment-related characteristics is collected from hospital records by data managers of IKNL. Information on treatment is restricted to interventions that are part of the (initial) primary treatment plan. Follow-up information on patients’ vital status is obtained through a yearly record linkage with the Municipal Personal Records Database (*Gemeentelijke Basisadministratie*, GBA), which was updated for this study until January 31st, 2023. Information on recurrent disease or tumour progression is not routinely collected in the DBTR/NCR and therefore was not available to us.

The study design was approved by the scientific committee of the DBTR. The data abstraction process and storage protocols for the study were approved by the supervisory committee of the NCR.

### Study population

**Fig. 1:**
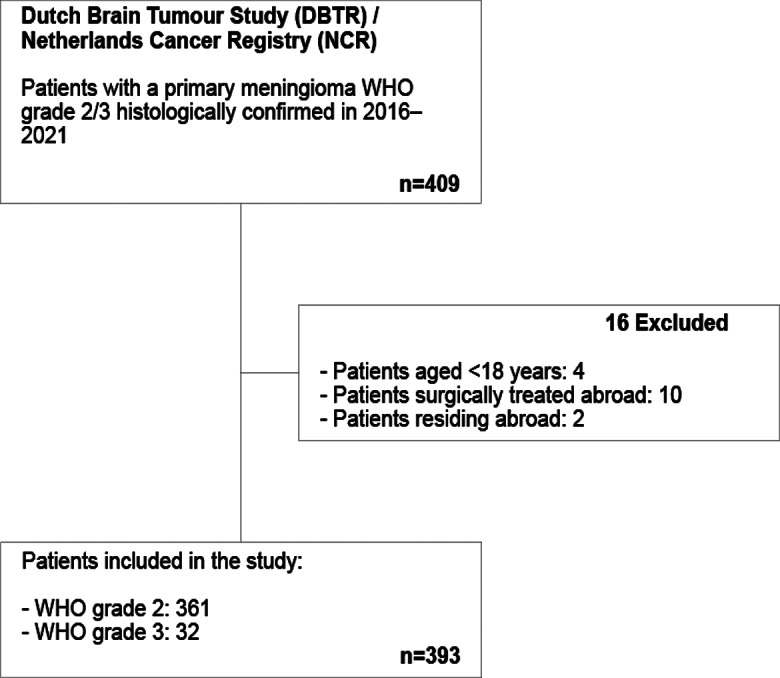
STROBE diagram for the selection of patients for the study. STROBE = Strengthening the reporting of observational studies in epidemiology.

We selected all Dutch patients with a histologically confirmed WHO grade 2–3 meningioma, diagnosed between 2016 and 2021, from the NCR/DBTR database (Fig. 1). We excluded patients < 18 years at diagnosis, those who were surgically abroad and those residing abroad. The resulting cohort included 393 patients, 361 with a grade 2 tumour and 32 with grade 3 disease.

Among the study variables available for analysis, patient characteristics included sex (male/female), age at diagnosis (18–59/60–74/≥75 years), postoperative Karnofsky performance score (KPS: <70/≥70), and socioeconomic status (low/medium/high). Socioeconomic status was based on the standardised median household income per residential postal code as determined in 2016 by Statistics Netherlands (*Centraal Bureau voor de Statistiek*, CBS). Tumour characteristics—aside from meningioma subtype and WHO grade—included presence of clinical symptoms prior to surgery (no/yes/not specified), tumour localisation (convexity/skull base/spinal/not specified), multiple lesions (no/yes), bone invasion (no/yes/not specified), and neural tissue invasion (no/yes/not specified).

Regarding primary treatments, we examined the extent of surgery (partial/total resection) and whether adjuvant RT was provided < 6 months following surgery. Simpson grades I–III constituted total resections, and Simpson grades IV–V were considered partial resections. If RT was provided ≥ 6 months, we classified initial management as no adjuvant RT. As neuro-oncology care in the Netherlands is largely organised in networks of hospitals centered around CNS regional tumour boards, which review individual patients’ diagnosis and formulate recommended treatment plans, we included these in our analyses.

### Statistical analysis

In describing the study cohort, we compared baseline and tumour characteristics between patients who received adjuvant RT < 6 months following surgery and those who did not with Chi-square tests and Fisher’s exact tests. Factors potentially associated with adjuvant RT were screened using the least absolute shrinkage and selection operator (LASSO) [17], and mutual associations between variables were summarised in a directed acyclic graph (DAG; Supplementary Fig. S1) [18, 19]. We confirmed associations through backward stepwise logistic regression analyses that included CNS regional tumour boards as a random effect, and our model selection was based on the Akaike information criterion (AIC). We presented effect estimates as odds ratios (ORs) together with 95% confidence intervals (CIs). For the definitive mixed-effect model, we reported the degree of clustering in provision of adjuvant RT through intraclass correlation coefficients (ICCs) and calculated the area under the (receiver operating characteristic) curve (AUC).

Variation between CNS regional tumour boards in providing adjuvant RT was further assessed by case-mix adjusted funnel plots [20–22]. These were constructed by plotting the observed-to-expected ratio of adjuvant RT against the total patient volume per tumour board during the study period. Expected ratios were calculated from individual patients’ expected values based on the logistic regression models that included factors identified by the abovementioned DAG (Supplementary Fig. S1), summed over all patients treated within each tumour board. Control limits were created at two and (95%) three (99.8%) standard errors from the target (observed/expected ratio of 1), and standard errors were derived under the assumption of a Poisson distribution. We presented separate plots for patients with a WHO grade 2 meningioma following a total resection, and for those with either a WHO grade 2 tumour following a partial resection or a WHO grade 3 meningioma.

To assess the benefit of adjuvant RT in our study population, we examined patients’ overall survival (OS) within 5 years following diagnosis. In the univariable setting, we applied the Kaplan-Meier method to present survival curves and assessed significant differences between patients with and without adjuvant RT using log-rank tests. Although we had intended to evaluate the independent effect of adjuvant RT on OS after adjustment for potential confounders under the assumption of proportional hazards, this assumption was considered violated for some subsets of patients, and we instead resorted to accelerated failure models with a Weibull distribution to estimate hazard rates (HRs) and survival functions.

To adjust for confounding, we applied inverse probability-weighting (IPW) in each subset of patients [23]. By weighting each individual in the analyses by the inverse probability of receiving adjuvant RT, we aimed to balance baseline characteristics between treated and untreated patients, thus approximating what would have been achieved through randomisation, although only for measured effects (i.e. pseudorandomisation). Weights were stabilised to account for extreme weights. Variable selection occurred on the basis of identified associations for each subset of patients as summarised in DAGs (Supplementary Fig. S2) [24].

For several variables, information was missing in patients, with postoperative KPS exhibiting a substantial proportion of missing data (46.3%). Complete information across all variables was obtained in 183 patients (46.6%). We assumed data to be missing at random, and observed no patterns of non-random missingness across analyses. To account for missing data, we performed analyses on datasets (m = 10) created through multiple imputation by chained equations (MICE) [25, 26]. The resulting estimates and standard errors were combined using Rubin’s rules [27]. For each analysis, we identified variables to be used in the imputation procedure by the DAGs, including the main outcome (adjuvant RT and OS, respectively) and CNS regional tumour boards (mixed-effect model). For comparison, we provided results from analyses restricted to complete cases along with those obtained following imputation.

All statistical analyses were two-sided, and we considered a p-value < 0.05 as significant. Analyses were performed using software package Stata version 17.0 (StataCorp, College Station, Texas) and we applied the web application Dagitty for analysing DAGs.

## Results

### Patient and tumour characteristics

Among 393 patients with a WHO grade 2–3 meningioma operated between 2016 and 2021, 361 were diagnosed grade 2 (91.9%), and 32 grade 3 (8.1%; Table 1). The majority was female (57.0%, *n* = 224), and most were below the age of 75 years (85.8%, *n* = 337). Among patients for whom postoperative KPS was reported, most were in good condition (111/136: 81.6%). Regarding tumour characteristics, most meningiomas were located at the convexity (62.8%, *n* = 247) followed by the skull base (24.7%, *n* = 97), and mostly concerned single lesions (92.1%, *n* = 362). Invasion of bone or neural tissue occurred in 10.4% (*n* = 41) and 24.7% (*n* = 97) of cases, respectively.

**Table 1:** Baseline and tumour characteristics of adult patients diagnosed with a WHO grade 2–3 meningioma in 2016–2021, and management by adjuvant RT < 6 months after surgery

	Total (*n* = 393)			Adjuvant RT (*n* = 139; 35.4%)			No adjuvant RT (*n* = 254; 64.6%)		
	*n*	%		*n*	%		*n*	%	*p*-value
*Sex*									0.64
Male	169	43.0%		62	44.6%		107	42.1%	
Female	224	57.0%		77	55.4%		147	57.9%	
*Age at diagnosis, years*									0.69
18–59	172	43.8%		63	45.3%		109	42.9%	
60–74	165	42.0%		59	42.4%		106	41.7%	
≥ 75	56	14.2%		17	12.2%		39	15.4%	
Median (IQR)	62 (49–71)			61 (52–71)			63 (48–71)		
Postoperative KPS*									0.28
< 70	32	8.1%		22	8.7%		10	7.2%	
≥ 70	179	45.6%		105	41.3%		74	53.2%	
Not specified	182	46.3%		127	50.0%		55	39.6%	
*Socioeconomic status*									0.39
Low	91	23.2%		27	19.4%		64	25.2%	
Medium	195	49.6%		74	53.2%		121	47.6%	
High	107	27.2%		38	27.3%		69	27.2%	
Clinical symptoms*									0.33
No	10	2.5%		5	3.6%		5	2.0%	
Yes	370	94.1%		130	93.5%		240	94.5%	
Not specified	13	3.3%		4	2.9%		9	3.5%	
*WHO grade*									< 0.01
Grade 2	361	91.9%		117	84.2%		244	96.1%	
Grade 3	32	8.1%		22	15.8%		10	3.9%	
Tumour localisation*									0.08**
Convexity	247	62.8%		87	62.6%		160	63.0%	
Skull base	97	24.7%		37	26.6%		60	23.6%	
Intraventricular	10	2.5%		0	0.0%		10	3.9%	
Spine	28	7.1%		11	7.9%		17	6.7%	
Not specified	11	2.8%		4	2.9%		7	2.8%	
*Multiple lesions*									0.71
No	362	92.1%		129	92.8%		233	91.7%	
Yes	31	7.9%		10	7.2%		21	8.3%	
Bone invasion*									< 0.01
No	308	78.4%		96	69.1%		212	83.5%	
Yes	41	10.4%		24	17.3%		17	6.7%	
Not specified	44	11.2%		19	13.7%		25	9.8%	
Neural tissue invasion*									0.87
No	252	64.1%		86	69.1%		166	65.4%	
Yes	97	24.7%		34	17.3%		63	24.8%	
Not specified	44	11.2%		19	13.7%		25	9.8%	
*Surgical procedure*									< 0.01
Partial resection	108	27.5%		66	47.5%		42	16.5%	
Total resection	285	72.5%		73	52.5%		212	83.5%	

* tested excluding ‘Not specified’ category

** Fisher’s exact test

RT = radiotherapy

IQR = interquartile range

KPS = Karnofsky Performance Score

WHO = World Health Organisation

### Adjuvant radiotherapy

Most meningiomas were resected in toto (72.5%, *n* = 285), while a partial resection took place in over a quarter of cases (27.5%, *n* = 108). Total resections were achieved in almost three-quarter of patients with a WHO grade 2 tumour (73.7%, *n* = 266), and under two-third of those with a WHO grade 3 meningioma (59.4%, *n* = 19). Surgical procedures were performed within twelve CNS regional tumour boards.

Overall, adjuvant RT < 6 months following surgery was provided in over one third of patients (35.4%, *n* = 139). Among patients with a WHO grade 2 meningioma confirmed after a total resection, just over one-fifth (22.2%, *n* = 59) received adjuvant RT (Table 2). Among those with a WHO grade 2 tumour following a partial resection, adjuvant RT was administered in 61.1% (*n* = 58) of cases. Adjuvant RT was provided to 68.8% (*n* = 22) of patients with a WHO grade 3 meningioma, 61.5% (*n* = 8) following a partial resection, and 73.7% (*n* = 14) following a total resection.

**Table 2:** Guideline recommendations of the European Association of Neuro-Oncology versus observed adjuvant RT for WHO grade 2–3 meningioma in the Dutch Brain Tumour Registry

	*n*	Recommendations for therapeutic management		Adjuvant RT	
				*n*	%
WHO grade 2					
Total resection	266	Watchful waiting or adjuvant RT		59	22.2%
Partial resection	95	Adjuvant RT		58	61.1%
WHO grade 3	32	Adjuvant RT		22	68.8%

RT = radiotherapy

WHO = World Health Organisation

Aside from higher tumour grade and subtotal surgical procedure, provision of adjuvant RT was associated with absence of multiple lesions and presence of bone invasion (Table 3). Associations with postoperative KPS and socioeconomic status did not prove statistically significant. The likelihood of receiving adjuvant RT varied across CNS regional tumour boards to some extent. Following mixed-effect modelling based on imputed data sets, the variance between tumour boards accounted for 14% (95%-CI: 5–32%) of the total variability in provision of adjuvant RT in the null (unconditional) model, and 13% (95%-CI: 4–31%) in the full (conditional) model. The AUC for the latter model was 79% (95%-CI: 72–85%).

**Table 3:** Mixed-effect multivariable logistic regression models exploring factors associated with adjuvant RT < 6 months after surgery for patients with a WHO grade 2–3 meningioma

	Complete case analysis (*n* = 183)			Analysis with imputed data sets (*n* = 393)		
	OR	(95%-CI)	*p*-value	OR	(95%-CI)	*p*-value
Postoperative KPS						
< 70	Ref			Ref		
≥ 70	1.90	(0.62–5.79)	0.26	2.11	(0.77–5.82)	0.14
*Socioeconomic status*						
Low	Ref			Ref		
Medium	1.62	(0.63–4.20)	0.32	1.31	(0.69–2.49)	0.41
High	2.46	(0.84–7.17)	0.10	1.41	(0.68–2.93)	0.36
*WHO grade*						
Grade 2	Ref			Ref		
Grade 3	6.30	(1.99–19.92)	< 0.01	4.34	(1.78–10.57)	< 0.01
*Multiple lesions*						
No	Ref			Ref		
Yes	0.77	(0.18–3.27)	0.72	0.33	(0.12–0.91)	0.03
*Bone invasion*						
No	Ref			Ref		
Yes	2.72	(0.98–7.55)	0.05	2.49	(1.03–6.00)	0.04
*Surgical procedure*						
Partial resection	Ref			Ref		
Total resection	0.16	(0.07–0.35)	< 0.01	0.17	(0.09–0.30)	< 0.01
ICC null model	0.14	(0.05–0.32)		0.14	(0.05–0.32)	
ICC CNS regional tumour boards	0.06	(0.01–0.48)		0.13	(0.04–0.31)	
AIC	216.59			431.07		
AUC	0.79	(0.73–0.86)		0.79	(0.72–0.85)	

RT = radiotherapy

OR = odds ratio

CI = confidence interval

WHO = World Health Organisation

ICC = intraclass correlation coefficient

CNS = central nervous system

AIC = Akaike information criterion

AUC = area under the (receiver operating characteristic) curve

**Fig. 2:**
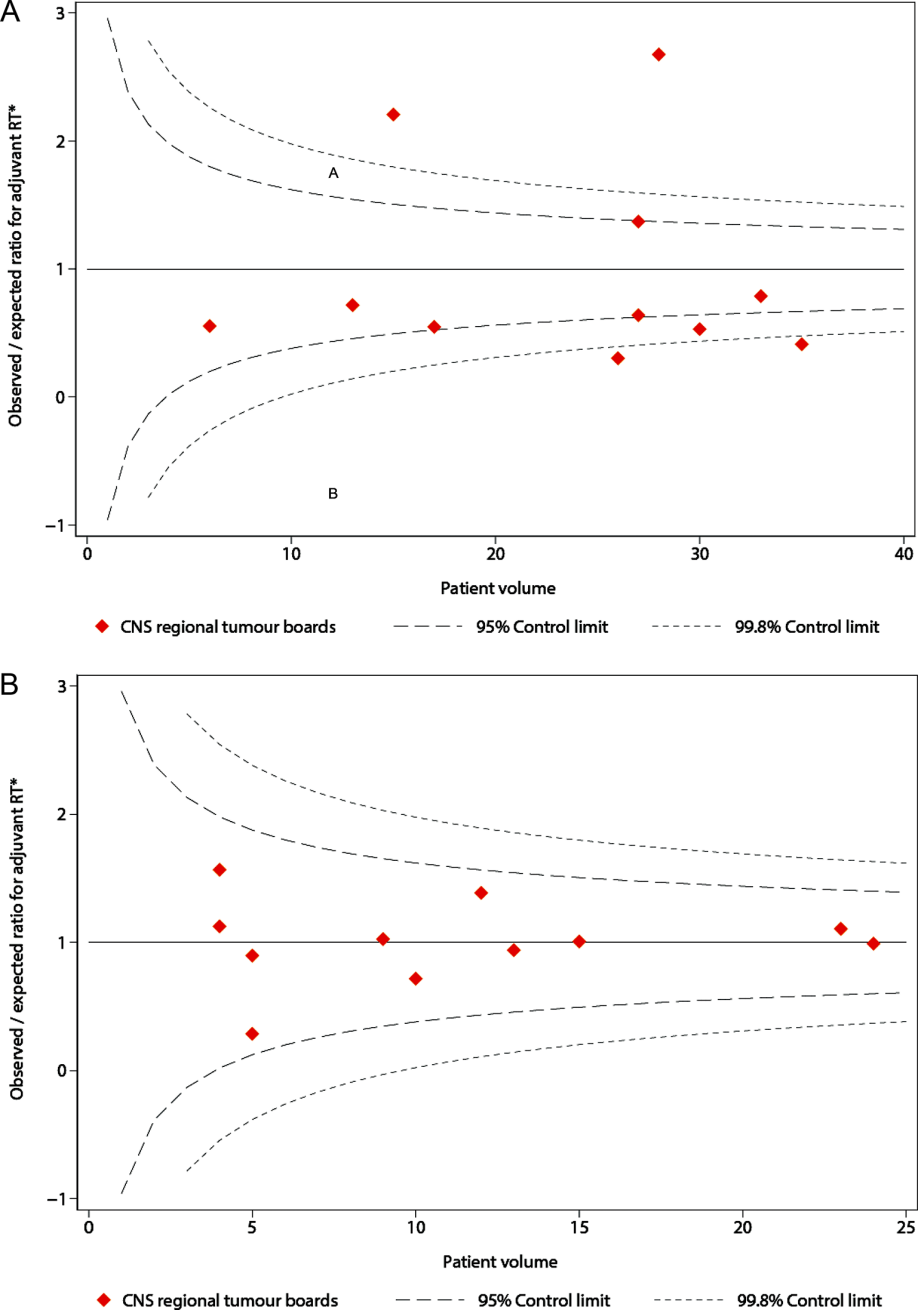
Funnel plots for observed-to-expected ratios in provision of adjuvant RT < 6 months after surgery across CNS regional tumour boards, for (A) patients with a WHO grade 2 meningioma following a total resection; (B) patients with a WHO grade 2 meningioma following a partial resection and patients with a WHO grade 3 meningioma. * Expected rates were based on the results of the mixed-effect multivariable logistic regression models including postoperative KPS, socioeconomic status, multiple lesions and bone invasion

Variability was observed for patients with a WHO grade 2 meningioma following a total resection (Fig. 2A). When comparing the observed rates of adjuvant RT within tumour boards to their expected rates based on their patients’ case mix, two tumour boards presented significantly higher ratios, scoring well above the upper 99.8% control limit of the case mix model. In contrast, two tumour boards scored just below the model’s lower 99.8% control limit. For patients with a WHO grade 2 meningioma following a partial resection and those with a WHO grade 3 tumour, provision of adjuvant RT took place within the 95% control limits of the case mix model across all tumour boards (Fig. 2B).

### Overall survival

**Fig. 3:**
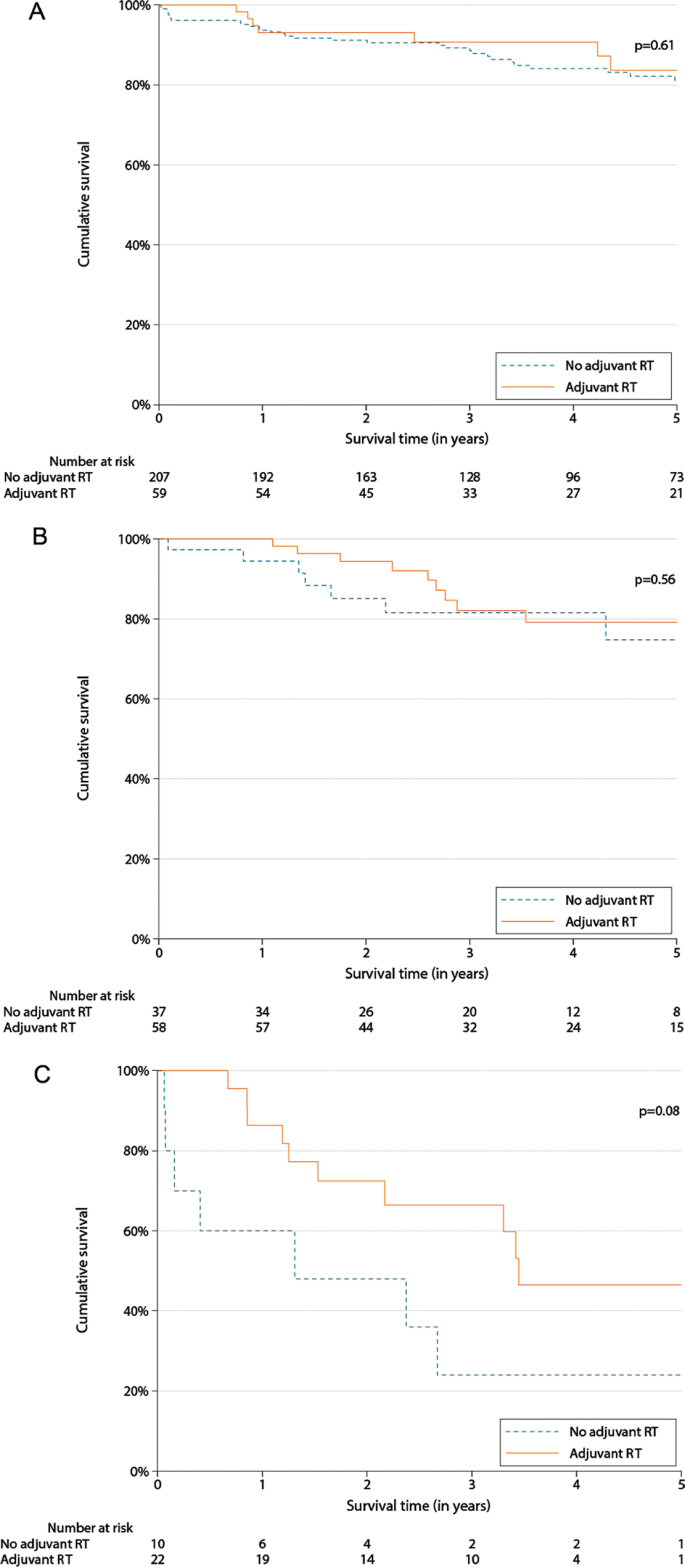
Kaplan–Meier curves representing overall survival according to adjuvant RT < 6 months after surgery, for (A) patients with a WHO grade 2 meningioma following a total resection; (B) patients with a WHO grade 2 meningioma following a partial resection; (C) patients with a WHO grade 3 meningioma. RT = radiotherapy, WHO = World Health Organisation

In univariable analyses, no differences in 5-year OS were detected between patients who received adjuvant RT < 6 months after surgery and those who did not (Fig. 3). Among patients with a WHO grade 2 meningioma following a total resection, OS after 5 years was 79.2% (95%-CI: 63.4–88.7%) for those who received adjuvant RT, and 74.8% (95%-CI: 51.9–88.0%) for those who did not (Fig. 3A). At 3 years, OS in those who received adjuvant RT was 90.7% (95%-CI: 79.0–96.1%). For patients following a partial resection, 5-year OS were 83.6% (95%-CI: 67.2–92.3%) and 81.1% (95%-CI: 73.8–86.6%), respectively (Fig. 3B). 3-Year OS was 82.1% (95%-CI: 67.1–90.7%) in those who received adjuvant RT. Although the OS curves of patients with a WHO 3 grade meningioma appeared to diverge from one another, this did not reach statistical significance (*p* = 0.08; Fig. 3C): for patients who received adjuvant RT, OS after 5 years was 46.5% (95%-CI: 22.5–67.5%), while this was 24.0% (95%-CI: 3.8–53.7%) for those who did not receive adjuvant RT. In the first, OS after 3 years was 66.4% (95%-CI: 41.7–82.6%).

**Fig. 4:**
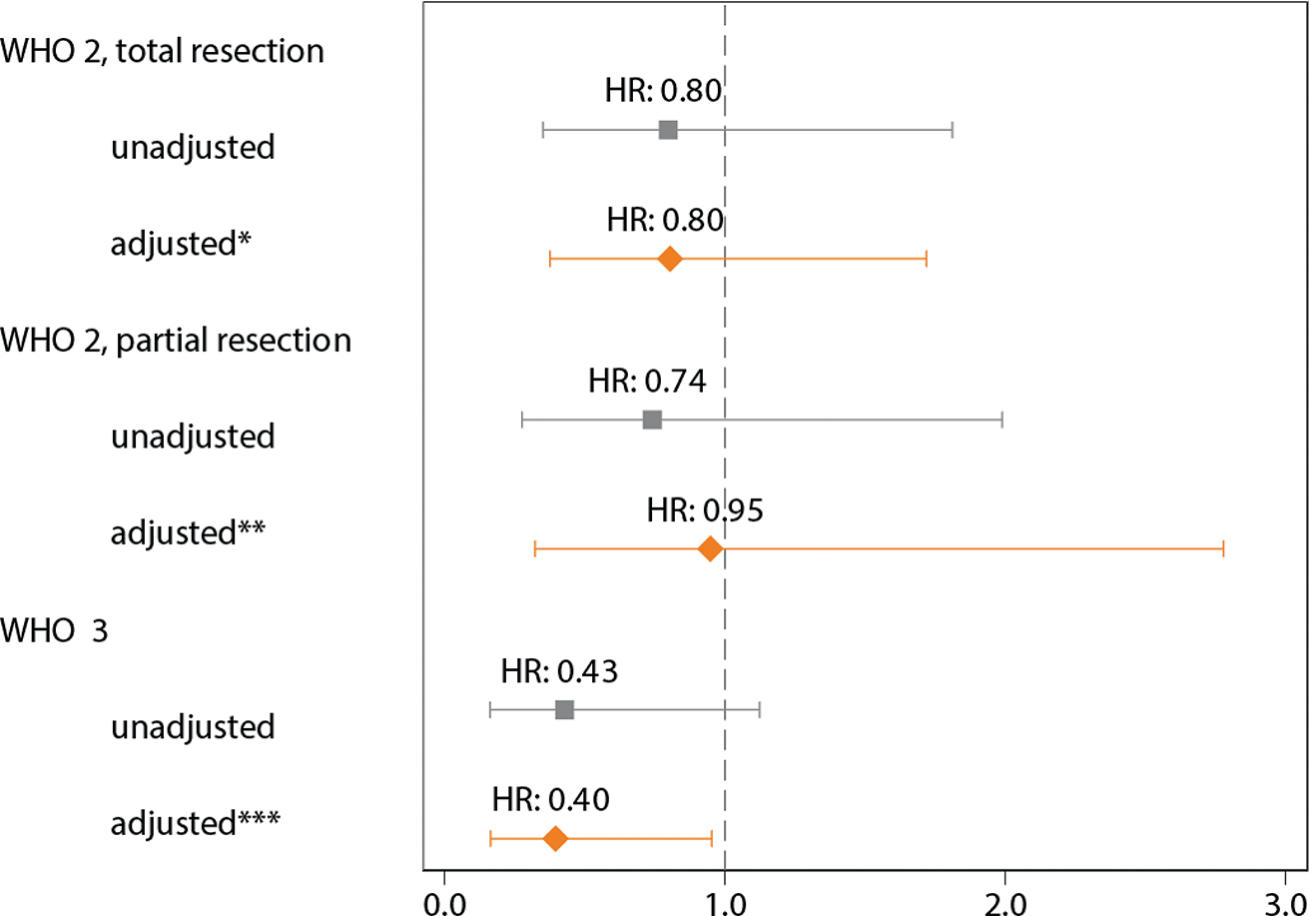
Overall survival hazard ratio (HR) estimates for the provision of adjuvant RT < 6 months after surgery in patients with a WHO grade 2–3 meningioma. IPW-adjustment with the following variables: * sex, age at diagnosis, postoperative KPS, ** sex, age at diagnosis, postoperative KPS, tumour localisation, multiple lesions, neural tissue invasion, *** sex, age at diagnosis, postoperative KPS, socioeconomic status, tumour localisation, neural tissue invasion. IPW = inverse probability weighting, RT = radiotherapy, HR = hazard ratio, KPS = Karnofsky Performance Score, WHO = World Health Organisation

Following adjustment for confounders, adjuvant RT < 6 months after surgery was associated with better OS only for patients with a WHO 3 grade meningioma (Fig. 4). While the unadjusted HR was 0.43 (95%-CI: 0.16–1.12), the adjusted HR was 0.40 (95%-CI: 0.16–0.95, *p* = 0.04).

## Discussion

This is the first study to report on the initial management of new WHO grade 2–3 meningioma in the Netherlands. Between 2016 and 2021, early adjuvant RT was provided to one-third of patients: while this proportion was lower among patients with an intermediate-risk tumour, the majority of those with high-risk disease received RT < 6 months after surgery. We could only demonstrate survival benefit of (early) adjuvant RT for patients with a WHO 3 grade meningioma.

No practice variation was observed for high-risk disease. Despite of the clear indication for adjuvant RT [3], a substantial proportion of patients in this subset did not receive treatment. This is understandable in case of WHO 3 grade meningioma, where lack of treatment is to an important degree due to patients’ poor prognosis (postoperative KPS appeared lower in those without adjuvant RT). As may be clear from the OS curves, early mortality prevented many of them from receiving treatment—a time-dependent bias that should also be considered in the association between OS and treatment [28]. In case of WHO grade 2 meningioma following partial resection, the reasons for withholding RT are less clear. We could not discern significant differences in either patient or tumour characteristics between patients who received treatment and those who did not.

Practice variation in the provision of adjuvant RT to patients with a new intermediate-risk meningioma was to be anticipated. In the absence of convincing evidence indicating a clear benefit, clinical decision making may depend more on individual doctors’ and patients’ judgments and preferences. Between hospitals—or networks of hospitals—, professional consensus may vary, among others, according to radiotherapists’ confidence in the potential of RT to achieve tumour control in this subset of patients, or in their ability to manage adverse effects once they occur. Indeed, two CNS regional tumour boards in this study showed a substantially higher proportion of treated patients than what was expected from our case mix model.

The OS rates found in our study were comparatively lower than those reported by the RTOG 0539 [6, 7] and EORTC 22,042 [8] trials. This is not surprising since our study population constituted an unselected patient cohort. Our findings in patients with a WHO grade 2 meningioma are largely in line with results obtained by other observational studies, most of which reported no significant effects of adjuvant RT on OS [9, 29]. OS in patients with a WHO grade 3 meningioma, on the other hand, compare unfavourably to what was found in a very large cohort over the period 2004–2014 [4]. However, coverage of that registry seemed less complete, and analyses were subsequently restricted to cases with complete information, which in conjunction may have strongly selected patients with better outcomes. A recently published multicentre study spanning 1991–2022 reported a 5-year OS of 66%, but a minimum follow-up time of 6 months following surgery was applied here for study inclusion [30].

Even though our study results do reflect actual practice patterns on a national level, their observational nature merits caution in interpretation. This is demonstrated, for instance, in the proportion of patients with missing information, particularly with respect to postoperative KPS. Even in case of substantial missingness, however, methods such as MICE should allow of valid effect estimations, provided that data are missing at random and the imputation models are properly specified [31].

Particularly with respect to our estimations of the effect of adjuvant RT, analyses could only include data routinely collected in the DBTR/NCR. Most importantly, we had no access to information on recurrent disease or tumour progression, nor on the intention with which treatment was provided. We therefore applied the 6 month time frame to distinguish adjuvant RT from RT given as salvage, postponed or palliative treatment. Although most meningioma recurrences are expected to occur within 2 years following surgery, probably depending on particular molecular subgroups [32], very rapid recurrences are rare [33]. Most series report 5-year recurrence rates of 30–60% for WHO grade 2 meningiomas [34, 35], and 60–90% for WHO grade 3 meningiomas [30, 36].

While some sources of potential confounding could presumably be corrected for by extending the dataset, others may not be so easily mitigated. We considered to ameliorate the abovementioned time-dependent bias—also known as ‘immortal time bias’—by applying a ‘landmark’ in analyses on patients with a WHO grade 3 meningioma. This would have restricted evaluation to cases who had survived a follow-up period long enough that they would have been able to receive adjuvant RT (landmark time). However, we deemed this method inviable due to the very small number of patients that would remain for analysis. Updated analyses should allow for larger sample sizes, also with respect to evaluating adjuvant RT in WHO 2 grade meningioma.

As our study demonstrated, registries such as the DBTR/NCR, despite of their limitations, have a role in monitoring practice patterns. Although the prospective ROAM/EORTC 1308 [13] and NRG-BN003 trials are expected to generate more evidence to guide clinical management, particularly for patients with a WHO grade 2 meningioma following a total resection, clinical decisions often deviate from recommended practices. By providing feedback as to which strategies are actually followed, registries may generate insight on how evidence is implemented and weighed against other considerations held by both doctors and patients in their shared decision-making. They may also facilitate doctors to compare their decisions, and thus learn from one another.

Evaluation of practice patterns should additionally prove insightful against the background of the recent changes implemented by the WHO classification [37]. By integrating molecular diagnostics with histopathological subtyping of meningiomas, the updated criteria introduced several genetic and epigenetic alterations associated with a higher risk of recurrence and worse prognosis, including *TERT* promoter mutations [38], homozygous deletion of *CDKN2A/B* [39], H3K27me3 loss of nuclear expression [40], and a hypermitotic DNA methylation profile [41]. In the coming years, it will become evident whether the anticipated shift in meningiomas towards high-risk disease will lead to an increased use of adjuvant RT.

In conclusion, this first national review on the management of WHO grade 2–3 meningioma constitutes a valuable addition to the evidence base beyond clinical trials. The study revealed variation in provision of adjuvant RT across hospital networks, but further analyses are needed to determine the reasons for this variation.

## Electronic supplementary material

Below is the link to the electronic supplementary material.

Supplementary Material 1

Supplementary Material 2

Supplementary Material 3

Supplementary Material 4

## Data Availability

The datasets generated during and/or analysed during the current study are available from the Netherlands Comprehensive Cancer Organisation (IKNL), Utrecht, the Netherlands, on reasonable request (https://iknl.nl/en/ncr/apply-for-data).
