# Supplementary figures and images for: Initial management of newly diagnosed WHO grade 2–3 adult meningioma following surgery: results from the Dutch Brain Tumour Registry (2016–2021)
Source: J Neurooncol. 2024 Aug 29;170(1):41–52. doi: 10.1007/s11060-024-04730-2 (PMC11446945; doi:10.1007/s11060-024-04730-2)

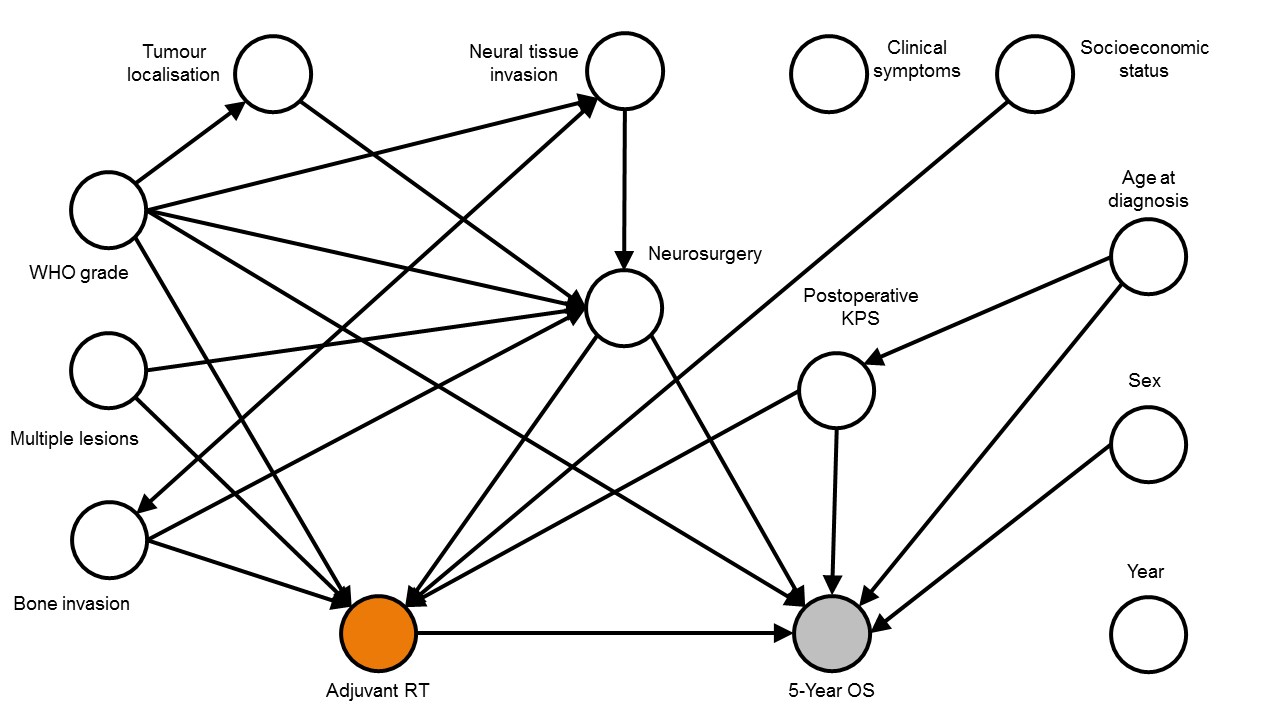

Supplement: Supplementary file 1 — Supplementary Material 1 [file 11060_2024_4730_MOESM1_ESM.jpg]

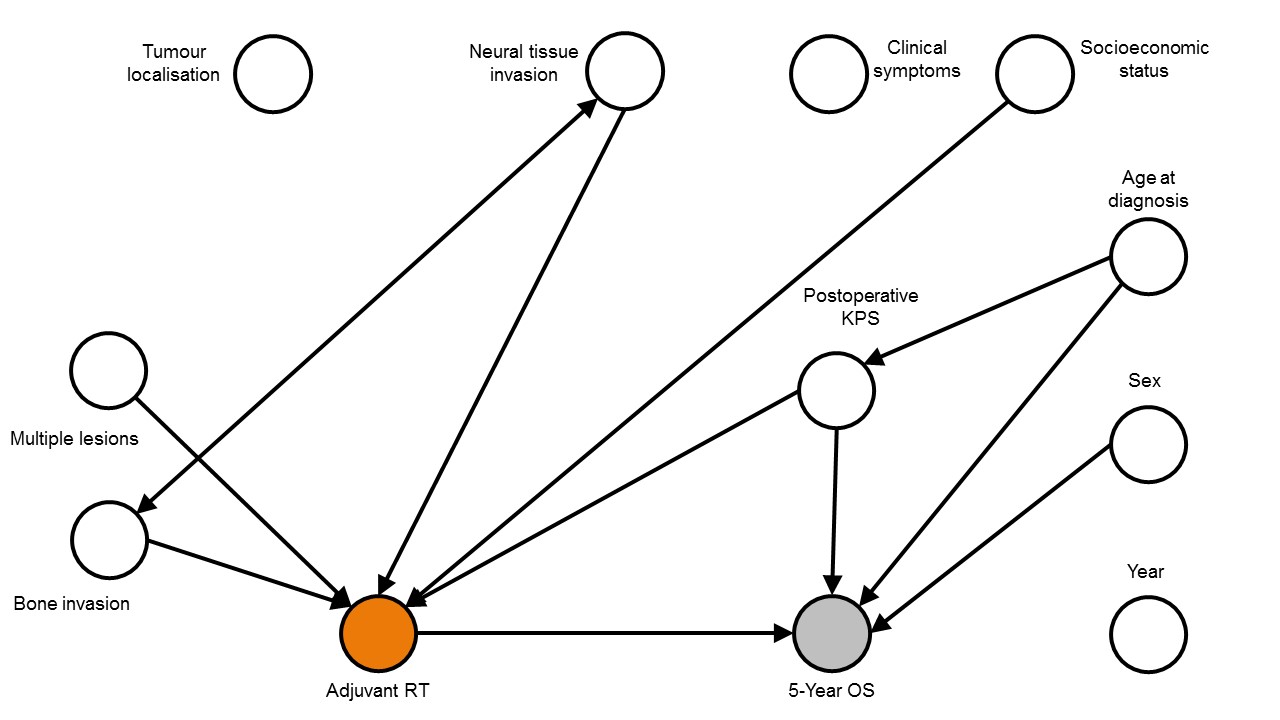

Supplement: Supplementary file 2 — Supplementary Material 2 [file 11060_2024_4730_MOESM2_ESM.jpg]

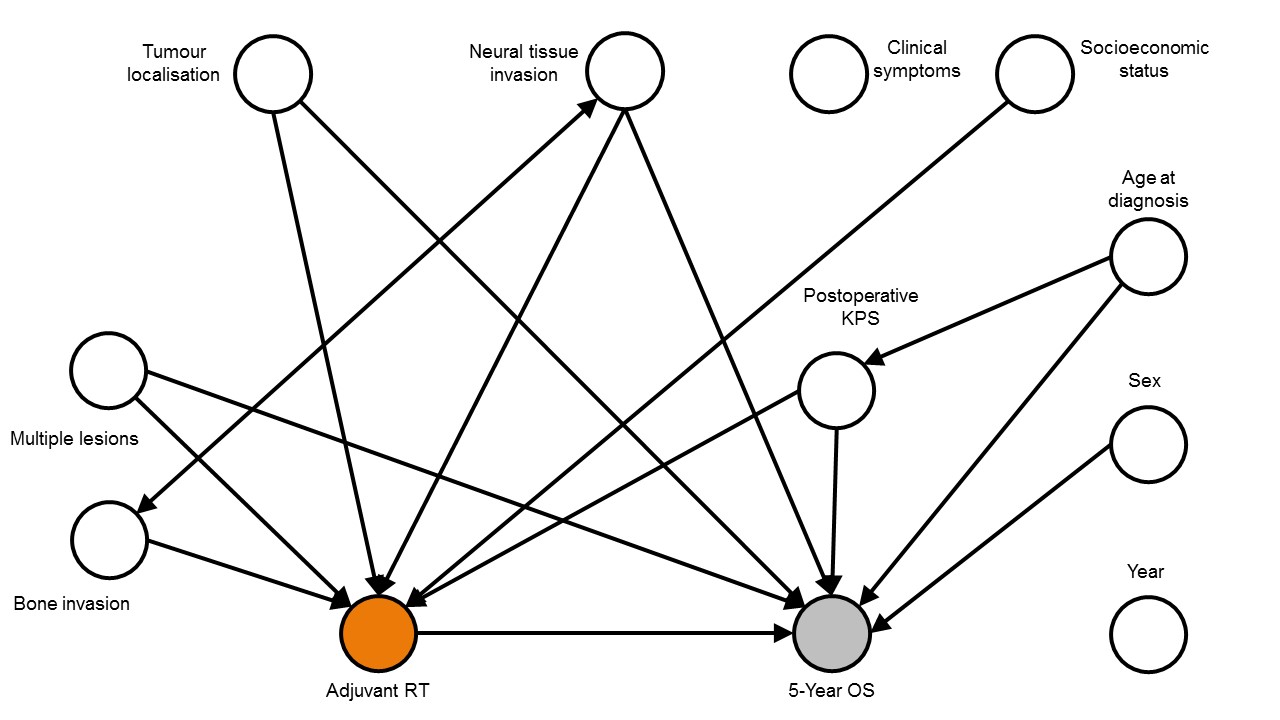

Supplement: Supplementary file 3 — Supplementary Material 3 [file 11060_2024_4730_MOESM3_ESM.jpg]

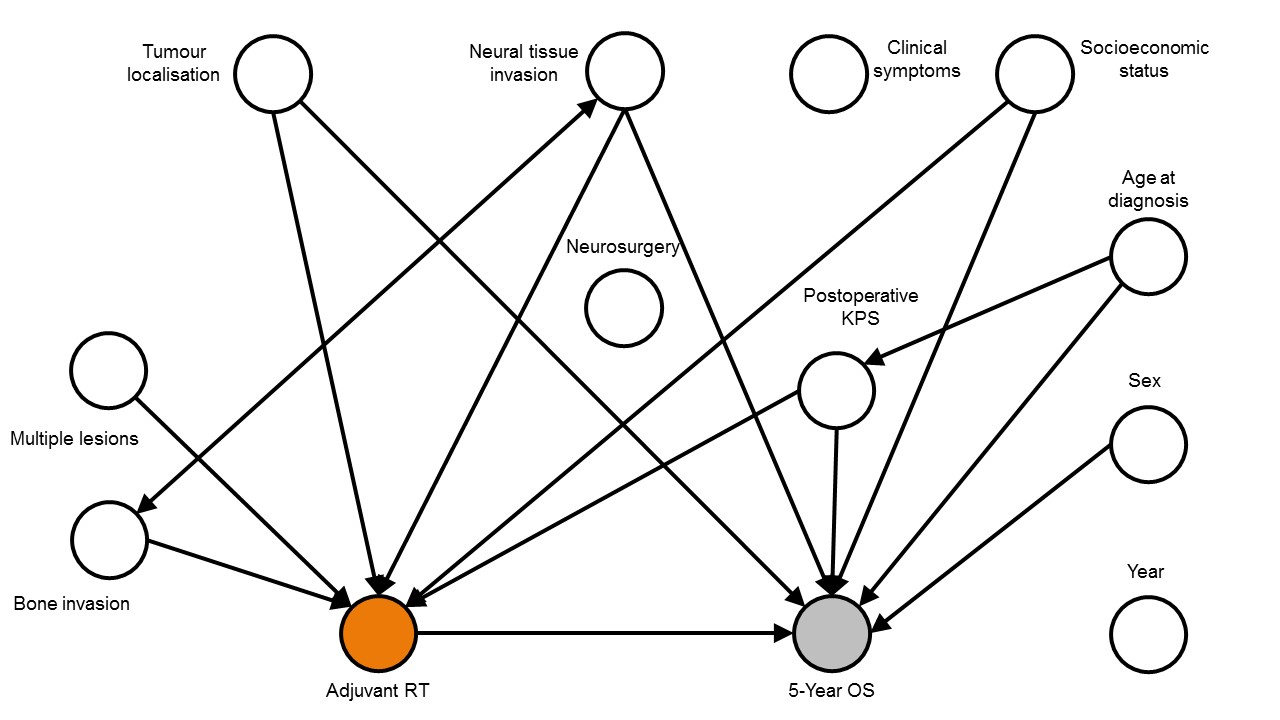

Supplement: Supplementary file 4 — Supplementary Material 4 [file 11060_2024_4730_MOESM4_ESM.jpg]
